# Soybean allergy: IgE epitopes of glycinin (Gly m 6), an important soybean allergen

**DOI:** 10.1186/2045-7022-4-S2-O2

**Published:** 2014-03-17

**Authors:** Dirk Schiller, Michael Hellmuth, Michaela Gubesch, Barbara Ballmer-Weber, Carsten Bindslev-Jensen, Bodo Niggemann, Joseph Scibillia, Kay-Martin Hanschmann, Yvonne Kühne, Andreas Reuter, Stefan Vieths, Andrea Wangorsch, Thomas Holzhauser

**Affiliations:** 1 Paul-Ehrlich-Institut, Division of Allergology, Langen, Germany; 2 Zurich University Hospital, The Allergy Unit, Department of Dermatology, Zurich, Switzerland; 3 Odense University Hospital, The Allergy Center, Odense, Denmark; 4 Charite University Hospital, Pediatric Pneumology, Berlin, Germany; 5 Niguarda Cá Granda Hospital, The Allergology and Clinical Immunology Unit, Milan, Italy; 6 Paul-Ehrlich-Institut, Department of Biostatistics, Langen, Germany

## Background

Soybean is an important food and known allergen. The hexameric major storage protein glycinin is an important soybean allergen (Gly m 6) but the knowledge about Gly m 6 IgE-epitopes is limited. Novel diagnostic and therapeutic approaches may be developed on the basis of known IgE epitopes.

## Objective

To identify IgE epitopes of Gly m 6 in a multi-peptide microarray.

## Methods

11 subjects with clinically confirmed soybean allergy and sIgE against rGly m 6 were enrolled in this study. Synthetic overlapping peptides (15-mers, 4 aa offset) representing the amino acid sequences of G1 and G5, two representative subunits of Gly m 6, were spotted onto CelluSpot™ slides and analyzed for IgE-binding, respectively. The sequences of IgE-binding peptides were allocated to the respective surface areas of the 3D-structures of G1 and G5. Allergen-specific IgE-binding to Gly m 6 peptides was verified by inhibition with rG1, rG5, and soy extract, respectively.

## Results

Gly m 6 peptides bound IgE of 100% (11/11) of the study sera. IgE-binding peptides covered 75% and 91% of the primary sequence of G1 and G5 subunits, respectively. Peptide-specific IgE-binding could be inhibited in a dose-dependent manner by rG1, rG5, and soy protein extract. 24 of G1 and 32 of G5 peptides bound sIgE by more than 25% of the sera. Allocation of these IgE-binding peptides to the surface of the structurally resolved Gly m 6 subunits showed a partial surface accessibility of the peptide sequences and yielded 15 and 18 putative IgE epitopes for G1 and G5, respectively. Comparison of the IgE-binding patterns in terms of severity of clinical symptoms or age showed a positive but statistically insignificant correlation.

## Conclusions

Allergen-specific multi-peptide microarray is a useful tool to identify IgE epitopes of food allergens. The identified putative IgE epitopes need to be further analyzed on the protein level. The knowledge of allergen-specific IgE epitopes allows the i) generation of hypoallergenic allergen variants for potential use as vaccine or therapeutic agent, ii) development of advanced diagnostic tools, and iii) assessment of the allergenicity of novel biotechnology products.

